# Biomarkers of Iron Are Associated with Anterior-Pituitary-Produced Reproductive Hormones in Men with Infertility

**DOI:** 10.3390/nu16020290

**Published:** 2024-01-18

**Authors:** Matineh Rastegar Panah, Keith Jarvi, Kirk Lo, Ahmed El-Sohemy

**Affiliations:** 1 Department of Nutritional Sciences, Temerty Faculty of Medicine, University of Toronto, Medical Sciences Building, 5th Floor, Room 5326A, 1 King’s College Circle, Toronto, ON M5S 1A8, Canada; matineh.panah@mail.utoronto.ca; 2 Murray Koffler Urologic Wellness Centre, Department of Urology, Mount Sinai Hospital, 60 Murray Street, 6th Floor, Toronto, ON M5T 3L, Canada

**Keywords:** male infertility, iron, ferritin, reproductive hormones, pituitary gland, luteinizing hormone, follicle-stimulating hormone, testosterone, estradiol, prolactin

## Abstract

Approximately 16% of North American couples are affected by infertility, with 30% of cases being attributable to male factor infertility. The regulation of reproductive hormones via the hypothalamic–pituitary–gonadal axis is important for spermatogenesis and subsequently male fertility. Maintaining iron homeostasis is critical to normal reproductive physiological function. This cross-sectional study’s objective was to determine the association between serum biomarkers of iron and reproductive hormones. Men experiencing infertility (*n* = 303) were recruited from Mount Sinai Hospital, Toronto. Serum was analyzed for iron and ferritin as biomarkers of iron status and reproductive hormones (follicle-stimulating hormone, luteinizing hormone, testosterone, estradiol, and prolactin), which were the primary outcome. Associations were determined using non-parametric Spearman’s rank correlation coefficient, linear regressions, and logistic regressions. A significant independent monotonic inverse relationship between serum iron and prolactin (*p* = 0.0002) was found. In linear regression analyses, iron was inversely associated with luteinizing hormone (unadjusted *p* = 0.03, adjusted *p* = 0.03) and prolactin (unadjusted *p* = 0.001 and adjusted *p* = 0.003). Serum ferritin was inversely associated with both gonadotropins, follicle-stimulating hormone (adjusted *p* = 0.03), and luteinizing hormone (adjusted *p* = 0.02). These findings suggest that biomarkers of iron are associated with pituitary-produced reproductive hormones, which play a role in the hypothalamic–pituitary–gonadal signaling pathway involved in spermatogenesis, testicular testosterone production, and male fertility.

## 1. Introduction

Infertility is defined as the inability to achieve conception after one year of unprotected intercourse [1]. In North America, nearly one in six couples are affected by infertility, with 30% of cases being caused by male infertility and another 20% of cases being caused by both male and female factor infertility [1]. With declining sperm quality and sperm quantity and an increase in hypogonadism, the prevalence of male infertility has been rising [2,3]. Previous research has found a significant age-independent population-level reduction in testosterone concentrations, which has been linked to possible health, nutritional, and environmental factors [2,4]. Male infertility has often been attributed to genetics, physical abnormalities, or endocrine disorders [5,6,7]. However, with growing rates of male infertility, recent studies have concentrated on the possible significance of lifestyle factors such as environmental toxin exposure, smoking, body weight, sleep disorders, diet, and nutrition [8,9,10,11,12,13,14,15]. Healthier dietary patterns have been linked to improved sperm parameters, but evidence of the effects of micronutrients on reproductive hormones is limited [16,17,18,19]. A recent review highlighted the potential significance of iron in the context of male reproductive health [20].

For the diagnosis of male factor infertility, seminal parameters and reproductive hormones serve as surrogate indicators [21,22]. The hypothalamic–pituitary–gonadal axis (HPG axis) is the primary signaling system responsible for regulating reproductive hormones, which play an important role in male fertility [23]. Gonadotropin-releasing hormone (GnRH), secreted by the hypothalamus, stimulates the release of gonadotropins, follicle-stimulating hormone (FSH), and luteinizing hormone (LH) in the anterior pituitary gland [23]. FSH acts on testicular Sertoli cells to promote several important functions essential to spermatogenesis, including sperm cell maturation [24]. LH acts on testicular Leydig cells to stimulate androgen synthesis, most notably testosterone, which is important for spermatogenesis [23,24,25]. Aromatase (CYP19) plays a role in facilitating the conversion of androgens to estrogens in peripheral tissues and testes, which can have negative effects on fertility, as elevated endogenous estrogen also inhibits the HPG axis via a negative feedback on the hypothalamus and pituitary gland [26,27]. Moreover, the HPG axis can also be inhibited by prolactin, a hormone produced by the anterior pituitary gland, resulting in the decreased synthesis of testosterone and reduced spermatogenic activity [28]. Maintaining hormonal balance is essential for promoting optimal male reproductive health.

Iron is an essential mineral with oxidative-reductive potential [29]. It plays an important role in preserving physiological functions, including oxygen transport, free radical homeostasis, the immune system, cognitive function, cellular metabolism, and enzyme function [30,31]. The mitochondrial reactions that produce the adenosine triphosphate that is responsible for promoting spermatogenesis and sperm motility require oxygen to function [32,33]. Iron deficiency anemia, a condition characterized by insufficient serum iron, is often associated with compromised circulatory oxygen transport in the testes leading to the creation of a hypoxic testicular environment [34]. Further, ferritin, the storage form of iron, is a significant secretory protein of Sertoli and Leydig cells and in animal models has been shown to protect testicular tissues [35,36]. On the opposing end, iron overload, a condition characterized by excess iron, can result in greater production of reactive oxygen species (ROS) and iron accumulation in tissues, ultimately leading to cellular death and impaired tissue function [29,37]. The pituitary gland is highly sensitive to iron deposition, as increased iron accumulation in the pituitary gland results in the destruction of gonadotrophs [29]. Additionally, iron overload is associated with reduced sperm motility, decreased ejaculate volume, increased abnormal sperm morphology, and diminished sperm motility [20]. The severe iron overload observed in hereditary hemochromatosis and β-thalassemia has been associated with infertility, sexual dysfunction, and hypogonadotropic hypogonadism in men [29]. Studies examining the role of iron biomarkers and reproductive hormones in humans have been limited [38]. Here, we examined the association between biomarkers of iron and reproductive hormones in an infertile male population.

## 2. Materials and Methods

### 2.1. Study Design and Participants

The study design is a cross-sectional analyses of serum iron biomarkers (iron and ferritin) and reproductive hormones in men with a male factor infertility diagnosis. Adult males (*n* = 832) experiencing infertility were recruited from Mount Sinai Hospital’s Murray Koffler Urologic Wellness Centre in Toronto between June 2019 and December 2021. The ineligibility criteria for study recruitment included individuals who had physical impairments causing infertility, were post-operative, or were unable to provide a venous blood sample. Those who had used fertility-related medication within the past six months, had a diagnosis for Klinefelter syndrome or cystic fibrosis, had undergone a vasectomy, or had undergone testicular cancer radiation therapy less than four years prior were excluded. After these exclusions, a total of 303 male participants were included in the analyses. The University of Toronto Research Ethics Board and Mount Sinai Hospital Research Ethics Board approved the study (14-0342-E, 5 February 2015), and all participants provided written informed consent to take part in the research.

### 2.2. Anthropometric Measurements and General Health Information

Participants completed a Mount Sinai Hospital’s Men’s Health Institute computerized personal health questionnaire. The questionnaire consisted of inquiries related to the participants’ anthropometric data (height and weight as self-reported), ethnic background, occupation, lifestyle, family history (including drug and alcohol use, smoking habits, etc.), fertility history, previous infertility treatments, urological history, andrology data, medication history, past medical and surgical history, and female partners’ fertility history. Participants also completed a sexual dysfunction and Sexual Health Inventory for Men (SHIM) questionnaire.

### 2.3. Nutritional and Reproductive Hormone Biochemical Measurements

Venous blood was collected and analyzed for serum iron biomarkers and reproductive hormones on-site at Mount Sinai Hospital’s laboratory or LifeLabs medical laboratory services. The serum reproductive hormones analyzed were FSH, LH, total testosterone (TT), prolactin, and estradiol, performed using ELISA assays as per the laboratory standard operating procedures. Due to laboratory reporting, estradiol levels below 93 pmol/L were reported as < 93 pmol/L.

### 2.4. Statistical Analysis

Statistical analyses were completed using R Studio (Version 1.1.463), where statistical significance was set at *p* < 0.05. For participants’ characteristics, simple descriptive analyses reported on the mean (±SD) and count (%) for continuous and dichotomous variables, respectively. Differences in categorical and continuous characteristics between tertiles of serum iron biomarkers were compared using chi-square and ANOVA, respectively. To evaluate the strength and direction of the independent monotonic relationship between serum iron biomarkers and reproductive hormones, non-parametric Spearman’s rank correlation coefficient analyses were employed. The normality of the data was assessed using the Shapiro–Wilk test. In the case of parametric analyses, the skewed distribution of reproductive hormones was normalized by square root transformations. Normal residual distribution was assessed for all models. The initial analyses examined the linear association between serum iron biomarker concentrations (iron and ferritin) and reproductive hormones using unadjusted and covariate-adjusted linear regressions. In the secondary analyses, logistic regressions were used. Both serum iron and ferritin were categorized into low, middle, and upper tertiles, while reproductive hormones were classified into categories of clinical significance. Tertiles were used for the categorization of serum iron and ferritin as opposed to the use of micronutrient deficiency and toxicity levels because the current biomarkers of iron status deficiency and toxicity cut-offs rely on disease-related epidemiological data and levels unrelated to fertility. The clinical cut-offs were based on reproductive hormones that were not within their normal ranges, such as elevated FSH (> 12.4 IU/L), elevated LH (> 7.8 IU/L), low TT (< 9.2 nmol/L), elevated prolactin (> 18 ng/mL), and elevated estradiol (> 146.8 pmol/L). The logistic regression analyses did not include prolactin due to the limited occurrence of elevated prolactin levels within the study population. Multivariate linear and logistic regression analyses were adjusted for several clinically relevant covariates including age in years, BMI in kg/m^2^, current alcohol consumption (yes/no/unspecified), smoking status, (yes/no), season (season (categorized according to meteorological seasons: ‘Winter’ from 1 December to 28/29 February, ‘Spring’ from 1 March to 31 May, ‘Summer’ from 1 June to 31 August, and ‘Fall’ from 1 September to 30 November), and ethnicity (categorized as Caucasian, African-Canadian, Asian, Hispanic, Indo-Canadian, Middle Eastern, and unspecified).

## 3. Results

The descriptive characteristics of participants by tertile of serum iron and ferritin are presented in Table 1. The mean ± SD iron and ferritin serum concentrations were 16.4 ± 5.4 μmol/L and 218 ± 161 μg/L, respectively. Iron deficiency, defined as a serum iron concentration below 11 μmol/L, was observed in 11.2% of the participants, while ferritin deficiency, defined as a serum ferritin concentration below 15 μg/L, was observed in 0.7% of the participants. Elevated ferritin, defined as a serum ferritin level greater than 300 μg/L, was present in 20.1% of participants. The mean age of the participants was 36.6 ± 5.8 years. The mean BMI was 27.9 ± 5.7 kg/m^2^, with 28.7% (*n* = 87) classified within the normal weight category (BMI < 25 kg/m^2^), 47.9% (*n* = 145) falling into the overweight category (BMI 25–29.9 kg/m^2^), and 23.4% (*n* = 71) within the obese category (BMI ≥ 30 kg/m^2^), corresponding to mean BMIs of 22.9 ± 1.4 kg/m^2^, 27.2 ± 1.3 kg/m^2^, and 35.5 ± 6.6 kg/m^2^, respectively.

The majority of the participants were Caucasian (44.6%), followed by Asian (19.1%), unspecified (14.2%), African-Canadian (8.9%), Middle Eastern (6.3%), Indo-Canadian (4.6%), and Hispanic (2.3%). Participants were predominantly non-smokers (85.8%) and non-alcohol consumers (53.5%). Low testosterone concentrations, defined as total testosterone concentrations below 9.2 nmol/L, were present in 30% (*n* = 91) of participants. Age (*p* = 0.02) and serum prolactin concentrations (*p* = 0.009) differed significantly across tertiles of serum iron concentrations (Table 1). There were no differences in participant characteristics across tertiles of serum ferritin concentrations.

Non-parametric Spearman’s rank correlation coefficient analyses, presented in Table 2, show a statistically significant independent monotonic inverse relationship between serum iron and prolactin (ρ = −0.22, *p* = 0.0002). No associations were observed between serum iron and any of the other reproductive hormones. No associations were observed between ferritin and any of the reproductive hormones. Non-parametric Spearman’s rank correlation coefficient analyses were not completed for estradiol due to the dichotomization of estradiol into normal (≤ 146.84 pmol/L) versus elevated (> 146.84 pmol/L) levels.

The results from the linear regression analyses (Table 3) showed that serum iron was inversely associated with LH (unadjusted model *p* = 0.03 and covariate-adjusted model *p* = 0.03) and prolactin (unadjusted model *p* = 0.001 and covariate-adjusted model *p* = 0.003). Furthermore, in the covariate-adjusted models, serum ferritin was associated with FSH (*p* = 0.03) and LH (*p* = 0.02). There was no significant linear relationship between ferritin or iron and the remaining reproductive hormones in either the unadjusted or adjusted analyses.

Logistic regression analyses examining the relationship between tertiles of serum iron biomarkers (reference group = lowest tertile) and clinical status of reproductive hormone (reference group = normal levels of reproductive hormones) are presented in Table 4. No significant associations were observed between the tertiles of iron and the clinical status of reproductive hormones. Furthermore, there were no significant associations between the tertiles of ferritin and the clinical status of reproductive hormones. Logistic regression analyses for prolactin were not conducted as the variability in elevated prolactin concentrations was less than 10%.

## 4. Discussion

To our knowledge, the present study is the first to examine the association between biomarkers of iron and reproductive hormones in a population of men with infertility. We found that serum iron and ferritin, as biomarkers of iron status, were inversely associated with reproductive hormones synthesized in the pituitary gland. An inverse relationship was observed between serum iron concentrations and both LH and prolactin, and serum ferritin concentrations exhibited an inverse association with both gonadotropins. An inverse linear association between serum iron and LH, but no significant association between serum iron and FSH, was observed. Previous research has shown the pituitary gland to be highly sensitive to iron, and gonadotrophs show progressively increasing iron deposition, especially in the fourth decade of life [29,39]. Evidence from MRI studies suggests that as iron stores increase in the body, there is a concomitant increase in iron deposition and decrease in pituitary volume [40,41]. Furthermore, in males with iron overload, autopsy studies have shown that iron deposition occurs in the gonadotrophs [42,43]. The underlying cause of hypogonadotropic hypogonadism observed in β-thalassemia and hereditary hemochromatosis is attributable to decreased LH synthesis. Previous research in men with β-thalassemia showed a 65% prevalence of hypogonadotropic hypogonadism [44]. Since iron overload adversely affects gonadotrophs, adverse effects on both LH and FSH synthesis is theoretically expected. However, a previous study with GnRH stimulation tests conducted on men with β-thalassemia found that FSH production is not as vulnerable as LH to iron accumulation [45]. This aligns with our findings, which indicate only an association between iron and LH but not FSH.

We also found an inverse linear association between serum iron and prolactin. Iron is essential for many biological functions, including the synthesis of neurotransmitters such as dopamine, which is the dominant negative regulator of prolactin [46,47]. Therefore, the inverse relationship observed between iron and prolactin may be mediated through the dopaminergic pathway. Clinically, these findings suggest that higher serum iron levels are associated with lower prolactin concentrations, which contribute to a more favorable hormonal environment for spermatogenesis and testosterone synthesis. Conversely, lower iron levels were associated with higher prolactin levels, which can lead to the inhibition of the HPG axis, consequently impacting testosterone levels. While the inverse relationship observed between serum iron and prolactin suggests a potential regulatory mechanism within the complex HPG axis signaling pathway, the clinical implications for male fertility are complex and warrant further investigation. The regulation of the HPG axis involves several multifaceted interactions. Furthermore, while an inverse association between iron and prolactin was observed, the absolute levels of prolactin might not be elevated to a degree that would unequivocally suppress the HPG axis and lead to a substantial reduction in testosterone. In addition, the clinical impact of prolactin on the HPG axis and testosterone levels often depends on the magnitude and duration of elevated prolactin.

No other significant associations were observed between iron and the remaining reproductive hormones (TT, estradiol, and prolactin). While the relationship between iron and reproductive hormones in the general population or population with infertility has not been assessed previously, a rodent model study found that mice fed iron-enriched diets had severe impairments to their HPG axis, leading to reduced LH and testosterone [48]. In human studies, there is some evidence to suggest that low testosterone concentrations are often seen in individuals with severe iron overload [29]. A study by Noetzli et al. found that pituitary iron concentrations are associated with testosterone concentrations in individuals with thalassemia [40]. However, it remains unclear whether the reduced testosterone concentrations in males with thalassemia are solely caused by decreased LH production or if the underlying cause is related to impairment in testicular testosterone production [29]. No studies have examined these associations in men with infertility. A recent study looking at ascorbic acid (vitamin C), a micronutrient that facilitates iron absorption, found that ascorbic acid was associated with favorable hormonal profiles in infertile males [49]. Ascorbic acid was inversely associated with LH and age-dependently positively associated with TT [49].

We also observed an inverse relationship between ferritin and both gonadotropins, LH and FSH. The limited number of studies on ferritin make it difficult to determine the underlying mechanism or make meaningful comparisons. Some previous research supports our findings, suggesting that high ferritin levels during puberty, particularly in thalassemia patients, are a risk factor for hypogonadotropic hypogonadism [50]. However, another cross-sectional study involving only 80 males with sickle cell anemia found that individuals in the high ferritin group had higher LH and lower testosterone in comparison to males in the normal ferritin group [51]. While we reported no association between ferritin and TT in infertile males, Liu et al. found a significant association between ferritin concentrations and TT after adjusting for age and alcohol consumption amongst healthy men [52]. In that study, however, they did not account for BMI as a covariate in their adjusted model when assessing the relationship between ferritin and TT, even though they observed a significant independent association between BMI and ferritin in their analyses [52]. Additionally, no association was observed in the present study between ferritin and either prolactin or estradiol.

In males with infertility, the prevalence of serum iron and ferritin deficiency, 11.2% and 0.7%, respectively, was not significantly different than expected in the general population. Ferritin deficiency in the study population was slightly lower than in the Canadian population data available from the Canadian Health Measures Survey (CHMS), where ferritin deficiency was observed in 1.4% of the male population aged 19 to 50 years. However, the CHMS reports a lower prevalence of ferritin deficiency at 0.8% in males in the age group of 51 to 79 years [53]. Elevated serum ferritin concentrations, which could be indicative of potential iron overload, were present in 20.1% of the study population. The prevalence of elevated ferritin levels is slightly above the 17.2% prevalence of elevated ferritin levels reported by the CHMS [53].

Although most of the research on iron and hormones has been conducted on individuals with severe iron overload, pituitary iron deposition is not exclusive to such conditions. Autopsy studies in the general population have revealed that gonadotrophs progressively accumulate iron deposits beginning in the fourth decade of life, leading to their eventual apoptosis and replacement with fibrosis. One of the notable strengths of our study is that it did not focus solely on iron overload, and we did not examine a population with severe iron overload, which is the predominant focus of previous research. Instead, our study focused on two iron biomarkers (iron and ferritin) and was carried out among a male infertile population. Other strengths of the present study include accounting for pre-determined covariates of clinical significance in the statistical analyses. Additionally, the use of iron biomarkers mitigated the risk of potential self-reporting bias, which is often a common concern with dietary intake assessment methods. The present study has some limitations. The cross-sectional nature of the study design precludes the establishment of causality and temporality. The risk of self-reporting bias for some of the covariates is a potential limitation of our study, as all demographic and anthropometric data were obtained through self-reports. While the study thoroughly examined the relationship between serum ferritin, serum iron, and reproductive hormones, the potential influence of inflammation on ferritin levels was not directly accounted for in the analysis. To advance our knowledge of the relationship between iron and male fertility, future research should examine these associations in diverse populations, followed by intervention studies in men with low iron status. Generating robust evidence using randomized controlled trials can help to further our understanding of this relationship and inform the development of effective interventions for male infertility. More evidence stemming from research in the field of nutrition and male infertility may yield various significant benefits, such as the integration of nutritional assessments and interventions to manage male infertility or to enhance the success rates of fertilizations through assisted reproductive technologies.

## 5. Conclusions

Iron uptake occurs in the pituitary gland, a critical site for reproductive hormone synthesis. However, no studies have examined the relationship between biomarkers of iron and reproductive hormones in infertile men. In the present study, serum iron concentrations were inversely associated with both LH and prolactin, while serum ferritin concentrations were inversely associated with both gonadotropins, FSH and LH. Our findings, indicating that biomarkers of iron status are associated with reproductive hormones synthesized in the anterior pituitary gland, suggest that optimizing iron stores could have beneficial effects on intratesticular testosterone production, sperm generation, and male reproductive health.

## Figures and Tables

**Table 1:** Subject characteristics by tertile of serum iron biomarkers.

Characteristics	Iron ^1^			*p* ^3^	Ferritin ^2^			*p* ^3^
	Lowest Tertile (*n* = 100)	Middle Tertile (*n* = 113)	Highest Tertile (*n* = 90)		Lowest Tertile (*n* = 101)	Middle Tertile (*n* = 100)	Highest Tertile (*n* = 102)	
Iron Biomarkers Serum Iron (μmol/L)	10.9 ± 2.0	16.1 ± 1.4	22.9 ± 3.6	-	15.0 ± 5.1	16.9 ± 5.3	17.3 ± 5.5	0.05
Iron clinical status ^4^, *n* (%) Sufficient Deficient	- - -	- - -	- - -	- - -	- 84 (83.2) 17 (16.8)	- 90 (90.0) 10 (10.0)	- 95 (93.1) 7 (6.9)	0.07 - -
Serum ferritin (μg/L)	184 ± 130	221 ± 165	252 ± 181	0.01	83.5 ± 30.4	184 ± 31.9	384 ± 169	-
Ferritin clinical status ^5^, *n* (%) Sufficient Deficient	- 98 (98.0) 2 (2.0)	- 113 (100) 0 (0.0)	- 90 (100) 0 (0.0)	0.13 - -	- - -	- - -	- - -	- - -
Factors of Clinical Relevance Age (years), mean ± SD	37.8 ± 6.1	36.5 ± 6.0	35.4 ± 4.9	0.02	36.5 ± 6.0	36.1 ± 5.5	37.2 ± 5.8	0.40
BMI (kg/m^2^), mean ± SD	28.5 ± 6.4	27.7 ± 5.5	27.5 ± 5.3	0.40	27.4 ± 4.9	27.3 ± 6.6	29.0 ± 5.5	0.52
Current Smoker	-	-	-	0.52	-	-	-	0.54
No	83 (83.0)	100 (88.5)	77 (85.6)	-	85 (84.2)	89 (89.0)	86 (84.3)	-
Yes	17 (17.0)	13 (11.5)	13 (14.4)	-	16 (15.9)	11 (11.0)	16 (15.7)	-
Current Alcohol Consumption	-	-	-	0.26	-	-	-	0.18
No	60 (60.0)	55 (48.7)	47 (52.2)	-	61 (60.4)	52 (52.0)	49 (48.0)	-
Yes	38 (38.0)	55 (48.6)	41 (45.6)	-	38 (37.6)	44 (44.0)	52 (51.0)	-
Unspecified	2 (2.0)	3 (2.7)	2 (2.2)	-	2 (2.0)	4 (4.0)	1 (1.0)	-
Ethnicity, *n* (%) Caucasian Asian Unspecified African Canadian Middle Eastern Indo-Canadian Hispanic	- 36 (36.0) 21 (21.0) 18 (18.0) 11 (11.0) 6 (6.0) 5 (5.0) 3 (3.0)	- 47 (41.6) 17 (15.0) 18 (15.9) 12 (10.6) 9 (8.0) 6 (5.3) 4 (3.5)	- 52 (57.8) 20 (22.2) 7 (7.8) 4 (4.4) 4 (4.4) 3 (3.3) 0 (0.0)	0.13 - - - - - - -	- 43 (42.6) 18 (17.8) 17 (16.8) 8 (7.9) 6 (5.9) 7 (6.9) 2 (2.0)	- 56 (56.0) 13 (13.0) 16 (16.0) 6 (6.0) 3 (3.0) 2 (2.0) 4 (4.0)	- 36 (35.3) 27 (26.5) 10 (9.8) 13 (12.7) 10 (9.8) 5 (4.9) 1 (1.0)	0.05 - - - - - - -
Seasonal variation ^6^, *n* (%)	-	-	-	0.98	-	-	-	0.82
Fall	33 (33.0)	36 (31.9)	27 (30.0)	-	33 (32.7)	34 (34.0)	29 (28.4)	-
Winter	29 (29.0)	35 (31.0)	26 (28.9)	-	31 (30.7)	28 (28.0)	31 (30.4)	-
Summer	28 (28.0)	32 (28.3)	25 (27.8)	-	24 (23.8)	28 (28.0)	33 (32.4)	-
Spring	10 (10.0)	10 (8.8)	12 (13.3)	-	13 (12.9)	10 (10.0)	9 (8.8)	-
Reproductive Hormones Serum TT (nmol/L)	12.8 ± 6.1	12.7 ± 6.9	14.4 ± 7.0	0.14	13.8 (7.8)	13.4 (4.9)	12.6 (7.0)	0.43
Serum FSH (IU/L)	10.6 ± 10.2	10.0 ± 9.9	8.7 ± 8.2	0.36	11.3 (11.5)	9.36 (8.6)	8.8 (8.1)	0.16
Serum LH (IU/L)	8.21 ± 6.1	7.0 ± 4.7	6.7 ± 4.01	0.08	8.2 (6.0)	6.6 (3.7)	7.0 (5.0)	0.06
Serum Prolactin (ng/mL)	9.5 ± 4.0	8.8 ± 3.7	7.9 ± 3.3	0.009	9.1 (3.7)	8.3 (3.3)	8.9 (4.1)	0.23
TT clinical status ^7^, *n* (%) Normal Low	- 70 (70.0) 30 (39.0)	- 72 (63.7) 41 (36.3)	- 70 (77.8) 29 (32.2)	0.09 - -	- 71 (70.3) 30 (29.7)	- 77 (77.0) 23 (23.0)	- 64 (62.7) 38 (37.3)	0.09 - -
FSH Clinical Status ^7^, *n* (%)	-	-	-	0.31	-	-	-	0.36
Normal	73 (73.0)	83 (73.5)	72 (80.0)	-	71 (70.3)	77 (77.0)	80 (78.4)	-
Elevated	27 (27.0)	30 (26.5)	18 (20.0)	-	30 (29.7)	23 (23.0)	22 (21.6)	-
LH Clinical Status ^7^, *n* (%) Normal Elevated	- 61 (61.0) 39 (39.0%)	- 80 (70.9) 33 (29.2)	- 61 (67.8) 29 (32.2%)	0.46 - -	- 61 (60.4) 40 (39.6)	- 71 (71.0) 29 (29.0)	- 70 (68.6) 32 (31.4)	0.25 - -
Prolactin clinical status ^7^, *n* (%) Normal Elevated	- 95 (95.0) 5 (5.0)	- 111 (98.2) 2 (1.8)	- 88 (97.8) 2 (2.2)	0.34 - -	- 99 (98.0) 2 (2.0)	- 98 (98.0) 2 (2.0)	- 97 (95.1) 5 (4.9)	0.37 - -
Estradiol clinical status ^7^, *n* (%) Elevated Normal	- 80 (80.0) 20 (20.0%)	- 84 (74.3) 29 (25.7%)	- 67 (74.4) 23 (25.6%)	0.56 - -	- 74 (73.3) 27 (26.7)	- 76 (76.0) 24 (24.0)	- 81 (79.4) 21 (20.6)	0.59 - -

^1^ Serum iron tertile cut-offs are ≤ 13 μmol/L, > 13 μmol/L to ≤ 18 μmol/L, and > 18 μmol/L for the lowest, middle, and highest tertiles, respectively. ^2^ Serum ferritin tertile cut-offs are ≤ 129.3 μg/L, > 129.3 μg/L to < 244 μg/L, and ≥ 244 μg/L for the lowest, middle, and highest tertiles, respectively. ^3^ Differences between groups were compared using chi-square and ANOVA for categorical and continuous variables, respectively. ^4^ Serum iron concentration cut-offs are < 11 μmol/L and ≥ 11 μmol/L for deficient and sufficient, respectively. ^5^ Serum ferritin concentration cut-offs are <15 μmol/L and ≥ 15 μmol/L for deficient and sufficient, respectively. ^6^ Seasonal variation was determined based on the meteorological season in which blood was drawn for analysis, ‘Winter’ = 1 December to 28/29 February; ‘Spring’ = 1 March to 31 May; ‘Summer’ = 1 June to 31 August; ‘Fall’ = 1 September to 30 November. ^7^ Clinical status cut-offs were based on reproductive hormones not within normal range: elevated FSH (> 12.4 IU/L), elevated LH (> 7.8 IU/L), low TT (< 265.3 ng/dL), elevated prolactin (> 18 ng/mL), and elevated estradiol (> 146.8 pmol/L).

**Table 2:** Spearman’s rank correlations for the independent association between serum iron biomarker concentrations and serum reproductive hormone concentrations.

Nutrient Biomarker	Hormone	ρ	*p*
Iron			
	FSH	−0.06	0.28
	LH	−0.09	0.10
	TT	0.10	0.09
	Prolactin	−0.22	0.0002
Ferritin			
	FSH	−0.03	0.61
	LH	−0.07	0.22
	TT	−0.12	0.05
	Prolactin	−0.04	0.44

Estradiol was excluded from the Spearman’s rank correlation analyses as estradiol was dichotomized into normal (≤ 146.8 pmol/L) and elevated (*>* 146.8 pmol/L) levels due to laboratory measurement sensitivity.

**Table 3:** Beta coefficient (± SE) and corresponding *p* for the linear association between serum iron biomarkers and serum reproductive hormone concentrations.

Nutrient Biomarker	Hormone ^1^	Unadjusted Model ß ± SE	Unadjusted *p*	Adjusted Model ß ± SE ^2^	Adjusted *p*
Iron					
	FSH	−0.02 ± 0.01	0.28	−0.004 ± 0.03	0.15
	LH	−0.02 ± 0.009	0.03	−0.02 ± 0.009	0.03
	TT	0.006 ± 0.01	0.51	0.004 ± 0.009	0.65
	Prolactin	−0.02 ± 0.006	0.001	−0.02 ± 0.006	0.003
	Estradiol ^3^	−0.02 ± 0.03	0.54	−0.03 ± 0.03	0.40
Ferritin					
	FSH	−0.0007 ± 0.0005	0.16	−0.001 ± 0.0005	0.03
	LH	−0.0004 ± 0.0003	0.16	−0.004± 0.002	0.02
	TT	−0.0005 ± 0.0003	0.09	0.0003 ± 0.0003	0.31
	Prolactin	−0.0002 ± 0.0002	0.37	−0.0002 ± 0.0002	0.21
	Estradiol ^3^	0.0003 ± 0.0009	0.71	0.0006 ± 0.001	0.51

^1^ Reproductive hormones (FSH, LH, TT, and prolactin) are square root transformed. The relationship depicted is the association between serum iron biomarker concentrations and the square root of reproductive hormones (FSH, LH, TT, and prolactin). ^2^ Model adjusted for potential covariates: age, alcohol consumption, BMI, ethnicity, seasonal variation, and smoking. ^3^ Estradiol was assessed categorically, with normal defined as levels ≤ 146.8 pmol/L and elevated defined as levels > 146.8 pmol/L).

**Table 4:** ORs and 95% CIs for the association between tertiles of serum iron biomarkers and reproductive hormones not within normal range ^1^ using binomial logistic regressions.

Hormone ^1^	Unadjusted OR [95% CI]	Unadjusted *p*	Adjusted OR [95% CI] ^4^	Adjusted *p*	Unadjusted OR [95% CI]	Unadjusted *p*	Adjusted OR [95% CI] ^4^	Adjusted *p*
	Serum Iron Middle Tertile ^2^				Serum Iron Highest Tertile ^3^			
FSH	0.98 [0.53, 1.79]	0.94	0.97 [0.51, 1.84]	0.92	0.68 [0.34, 1.33]	0.26	0.69 [0.33, 1.43]	0.32
LH	0.65 [0.36, 1.14]	0.13	0.68 [0.34, 1.39]	0.21	0.74 [0.41, 1.35]	0.33	0.82 [0.38, 1.76]	0.55
TT	1.33 [0.75, 2.36]	0.33	1.50 [0.80, 2.83]	0.21	0.67 [0.35, 1.28]	0.23	0.78 [0.38, 1.61]	0.50
Estradiol	0.72 [0.38, 1.38]	0.33	0.74 [0.37, 1.46]	0.38	0.73 [0.37, 1.44]	0.36	0.63 [0.30, 1.32]	0.22
	Serum Ferritin Middle Tertile ^2^				Serum Ferritin Highest Tertile ^3^			
FSH	0.69 [0.33, 1.33]	0.32	0.97 [0.34, 1.30]	0.23	0.65 [0.34, 1.23]	0.19	0.69 [0.27, 1.09]	0.09
LH	0.62 [0.35, 1.12]	0.11	0.63 [0.30, 1.30]	0.13	0.70 [0.39, 1.24]	0.22	0.60 [0.29, 1.25]	0.10
TT	0.71 [0.38, 1.33]	0.28	0.73 [0.36, 1.44]	0.36	1.41 [0.78, 2.52]	0.26	1.31 [0.68, 2.52]	0.42
Estradiol	1.16 [0.61, 2.18]	0.66	1.09 [0.55, 2.16]	0.70	1.41 [0.73, 2.70]	0.30	1.81 [0.89, 3.68]	0.11

^1^ Clinical status cut-offs were based on reproductive hormones not within normal range, defined as low TT (<9.2 nmol/L), elevated LH (>7.8 IU/L), elevated FSH (>12.4 IU/L), and elevated estradiol (>146.8 pmol/L). The lowest tertile of serum iron biomarkers and serum reproductive hormones within normal range was used as the reference category for all analyses. ^2^ ORs compare the odds of experiencing reproductive hormones not within normal range for participants in the middle tertile of iron biomarker concentrations with participants in the lowest tertile of iron biomarker concentrations. ^3^ ORs compare the odds of experiencing reproductive hormones not within normal range for participants in the highest tertile of serum iron biomarker concentrations with the odds for those in the lowest tertile of serum iron biomarker concentrations. ^4^ Model adjusted for potential confounders: age, BMI, ethnicity, smoking, seasonal variation, and alcohol consumption.

## Data Availability

The data presented in this study are available on request from the corresponding author. The data are not publicly available due to privacy restrictions.
